# Biallelic *MCUR1* nonsense mutation associated with vacuolar myopathy and altered mitochondrial calcium signaling

**DOI:** 10.1186/s40478-026-02313-y

**Published:** 2026-05-05

**Authors:** Anna Maria Haschke, Anja von Renesse, Eugenio Graceffo, Susanne Morales-Gonzalez, Alessandro Prigione, Christoph Hübner, Werner Stenzel, Markus Schuelke

**Affiliations:** 1 Charité-Universitätsmedizin Berlin, Corporate Member of Freie Universität Berlin, Humboldt-Universität zu Berlin, Berlin Institute of Health, NeuroCure Cluster of Excellence, 10117 Berlin, Germany; 2 Department of Neuropediatrics, Charité-Universitätsmedizin Berlin, Corporate Member of Freie Universität Berlin, Humboldt-Universität zu Berlin, Berlin Institute of Health, Augustenburger Platz 1, 13353 Berlin, Germany; 3 Department of General Pediatrics, Neonatology and Pediatric Cardiology, University Hospital Düsseldorf, Heinrich Heine University, 40225 Düsseldorf, Germany; 4 Department of Neuropediatrics, Universitätsklinikum Carl Gustav Carus, 01307 Dresden, Germany; 5 Berlin Institute of Health, Institute of Neuropathology, Charité-Universitätsmedizin Berlin, Corporate Member of Freie Universität Berlin, Humboldt–Universität zu Berlin, Charité-Universitätsmedizin Berlin, 10117 Berlin, Germany

**Keywords:** Mitochondrium, Mitochondrial calcium uniporter, Calcium transport, Autophagic vacuoles, Vacuolar myopathy

## Abstract

During muscle contraction, increased influx of calcium from the myocyte cytosol into the mitochondrial matrix through the mitochondrial calcium uniporter (MCU) links calcium homeostasis with high ATP provision. The MCU is located at the inner mitochondrial membrane and one of its structural components, the mitochondrial calcium uniporter regulator 1 (MCUR1), promotes its activity. Although MCUR1 function has been studied in cell models, mutations have not yet been associated with human disease. Here, we present a patient with proximal muscle weakness and atrophy, showing histological features of autophagic vacuoles with sarcolemmal features, who carries a homozygous *MCUR1* nonsense mutation. To investigate the underlying mechanisms of muscle pathology, we examined patient fibroblasts and quadriceps muscle specimens. MCUR1 deficiency compromised mtCa^2+^ uptake, that had been stimulated both by histamine or rising extracellular calcium exposure. Autophagic flux and histologic markers for autophagy (LAMP2, LCB3) were increased in the patient. However, the *MCUR1* mutation did not alter MCU-complex assembly or its subcellular location, nor the resting mitochondrial membrane potential. Our study associates MCUR1 deficiency with mitochondrial dysfunction and autophagic vacuolar myopathy, thereby highlighting the crucial role of mtCa^2+^ uptake in regulating mitochondrial function and expanding the spectrum of mitochondrial disorders in humans.

**Supplementary Information:**

The online version contains supplementary material available at 10.1186/s40478-026-02313-y.

## Introduction

Muscle contraction is a highly orchestrated process, by which calcium transients are released from the sarcoplasmic reticulum (SR) into the cytosol of the myocyte to activate its contractile elements. Mitochondria play a crucial role in regulating and moderating these calcium transients, mainly by removing the cytosolic Ca^2+^ ions and transporting them via the mitochondrial calcium uniporter (MCU) through the mitochondrial membrane into the mitochondrial matrix. From there, calcium is extruded back to the cytosol *via* NCLX a Sodium/Potassium/Calcium Exchanger (syn. SLC24A6) [5]. Due to the close spatial coupling between mitochondria and SR, the calcium ions are quickly syphoned up by the SERCA pumps into the SR [27]. This movement of Ca^2+^ ions is closely coupled with the oxidative phosphorylation that generates the negative mitochondrial membrane potential (MMP) and allows ATP production by the F_1_F_0_-ATPase. The highly negative voltage gradient across the inner mitochondrial membrane (IMM), known as the mitochondrial membrane potential (ΔΨ_M_), drives the mtCa^2+^ uptake. Under physiological conditions, calcium buffering through the mitochondria is central for ATP production, redox balance, and regulation of autophagy [13]. The mtCa^2+^ uniporter complex consists of three core pore-forming proteins [associated *gene* *symbols *], MCU [*MCU*], MCUb [*MCUB*], and EMRE [*SMDT1*] and of additional regulatory subunits (MICU1 [*MICU1*], MICU2 [*MICU2*], MICU3 [*MICU3*], and MCUR1 [*MCUR1*]) [1, 7, 8].

In 2013, Pan et al. generated an *MCU*^−/−^ mouse model by placing a gene trap into *MCU* intron 1. Homozygous mutant animals had a reduced body size and exhibited exercise intolerance. Isolated muscle mitochondria failed to take up calcium from the surrounding medium [21]. Other studies silenced the *MCU* gene with short hairpin RNAs (shRNA) that had been delivered by adeno-associated virus serotype 9 (AAV9) and confirmed these initial findings. *MCU* knock-down animals had muscle atrophy, distorted mitochondrial shapes, and an increased number of vacuoles. In contrast, *MCU* overexpression *via* AAV9-mediated gene transfer led to muscle hypertrophy [3, 18]. To date, no primary pathogenic mutations in the coding region of the core pore-forming subunit *MCU* have been conclusively linked to a human genetic disease.

However, researchers discovered recessive mutations in the MICU1 regulatory subunit. Patients suffered from early-onset proximal muscle weakness and intellectual disability [9, 16]. Another regulatory component of the MCU complex is the mitochondrial calcium uniporter regulator 1 (MCUR1), which is an integral membrane protein of the IMM. MCUR1 acts as a scaffold protein and promotes MCU activity. Its binding to both MCU and EMRE facilitates the assembly and stability of the MCU complex. Abrogation of MCUR1 in mouse cardiomyocytes and in endothelial cells disrupts the oligomerization of the MCU complex, underlining its importance in forming an active MCU complex [28]. Functional studies in HEK293T and HeLa cells have shown that *MCUR1* knock-down by RNA interference (RNAi) resulted in decreased mitochondrial Ca^2+^ uptake and basal oxygen consumption, as well as impaired oxidative phosphorylation (OXPHOS) and ATP production. This aligns with the effects observed in *MCU* knockout models [17, 29]. The findings support the hypothesis that MCUR1 is critical for MCU-mediated mtCa^2+^ uptake.

So far, *MCUR1* mutations have not yet been associated with human genetic disease. Here we present the case of a patient with a homozygous recessive nonsense mutation in *MCUR1*, and describe his muscle histology, calcium signaling, and effects on downstream regulatory pathways that are essential for maintaining muscle health.

## Materials and methods

### Consent

Written informed consent was obtained from the family of the patient. The study was approved by the institutional review board of Charité (EA2/202/08). All participants gave written informed consent in accordance with the Declaration of Helsinki for all aspects of the study. Anonymized control fibroblast lines and muscle biopsy specimens were obtained from leftover diagnostic samples from patients without neuromuscular disorders.

## Immunofluorescence staining

Fibroblast cells were seeded onto glass coverslips and cultivated at 37 °C and 5% CO_2_ until they reached 80% confluence. The mitochondria were stained with 250 nM MitoTracker Red (Thermo Fisher, M7512) in Opti-MEM (Reduced Serum Medium, Thermo Fisher, 31985-062) for 30 min under growth conditions. The cells were then fixed with a 4% PFA solution in PBS for 15 min and rinsed three times with PBS. For permeabilization, cells were incubated for 10 min with 0.1% Triton X-100 (Merck, 108643) in PBS under continuous shaking. After blocking with 10% normal goat serum (Abcam, ab7475) in PBS for 1 h, the cells were incubated with a monoclonal goat anti-MCU antibody (D2Z3B clone, dilution 1:100 dilution, Cell Signaling, #14997) overnight at 4 °C. After washing, the cells were incubated with the secondary antibody (goat anti-rabbit Alexa Fluor 488, Thermo Fisher A3273, dilution 1:200 with 10% normal donkey serum) for 45 min at RT under light protection. Counterstaining of the nuclei was done with DAPI (dilution 1:10,000; Invitrogen, ​​D1306). Cells were mounted on Mowiol 4–88 Mounting Media (ROTH, 0713). After immunostaining, cells were imaged with a Leica Thunder DMi8 microscope.

## Western blotting

Cells were lysed in RIPA lysis buffer [1% NP-40, 0.1% SDS, 50 mM Tris-HCl pH 8, 150 mM NaCl, 0.5% sodium deoxycholate, and Complete™ EDTA-free protease inhibitor (Roche, 11 873 580 001)] and quantified with the Protein Assay Dye Reagent Concentrate (Bio-Rad, #5000001). Protein was separated on a NuPAGE 4–12%, Bis-Tris gel (Thermo Fisher, NP0329), transferred to nitrocellulose membrane (Amersham, #10600001) and stained with Ponceau solution. Protein bands were visualized using specific antibodies (see Supplemental Table S2).

## Histology and histochemistry

All histological and histochemical studies were performed on cryosections of the quadriceps muscle. Histological stainings were imaged with a Leica DMLB microscope. For morphometric analysis, the muscle fibers’ Ferret diameter was determined with the ImageJ v1.45 software. For electron microscopy, skeletal muscle samples were fixed in 2.5% glutaraldehyde in 0.1 M sodium cacodylate buffer for 48 h at 4 °C. Samples were post-fixed in 1% osmium tetroxide in 0.05 M sodium cacodylate buffer for 3 h, dehydrated in graded acetone series including *en bloc* staining with 1% uranyl acetate and 0.1% phosphotungstic acid in the 70% acetone step for 60 min and embedded in araldite resin. Ultrathin sections were stained with uranyl acetate and lead citrate and imaged with a Zeiss P902 electron microscope [19].

## Functional assessment of patient material

### Oxygen consumption rate

The oxygen consumption rate (OCR) was measured with a Seahorse™ XF HS Mini Analyzer (Agilent) and XF Cell Mito Stress Test Kit (Agilent, #103010). 10,000 cells/well were seeded in a 8-well Seahorse™ XF Cell Culture Microplate (Agilent, #103725) and incubated overnight at 37 °C. Prior to the assay, growth media was replaced with XF DMEM medium, pH 7.4 (Agilent, #103575) supplemented with 1 mM sodium pyruvate (Sigma-Aldrich, #S8636) 2 mM L-glutamine (Gibco, #25030149) and 10 mM glucose (Sigma-Aldrich, #49163). Cells were then incubated for 45–60 min in 37 °C without CO_2_ to prevent acidification of the medium. The basal respiration rate was measured before cells were exposed sequentially to oligomycin (1 µM), carbonyl cyanide p-trifluoromethoxyphenylhydrazone (FCCP 1.5 µM) and rotenone + antimycin A (500 nM each). After each injection, the OCR was measured for 5 min, the medium was mixed and again measured for another 5 min. After the experiment, the cell number was determined by CyQUANT (Invitrogen, #C35011) for normalization.

## Assessment of live-cell ATP production

Fibroblasts were detached by 0.05% Trypsin-EDTA (Gibco, #25300054) treatment and pcDNA3.2-V5-mtLuc (Addgene, #219666) was introduced using Amaxa Nucleofector 2 (Lonza) and the Amaxa Human Dermal Fibroblast Nucleofector Kit (Lonza, VPD-1001) according to protocol. This vector targets the firefly luciferase into the mitochondrial matrix. In the presence of luciferin, the mitochondria emit light whose intensity is proportional to the ATP concentration in the mitochondrial matrix. After addition of 1 µM cell permeable DMNPE-caged Luciferin (Abcam, ab145163) we measured bioluminescence using the GloMax™ plate reader (Promega, GM3000). Data was normalized to the number of cells evaluated by CyQUANT™ (Invitrogen, #C35011).

## Intracellular Ca^2+^ imaging

Cells were transfected using Nucleofector as described before using the plasmids pCMV_R-GECO1 (Addgene #32444, measures intra-cytosolic Ca^2+^, red fluorescence) and pCMV_CEPIA2mt (Addgene #58218, measures intra-mitochondrial Ca^2+^, green fluorescence). For all live cell experiments, the cells were seeded in a m-Dish 35 mm with glass bottom (ibidi, 81158), mounted on a microscope stage and continuously perfused using a peristaltic perfusion pump (PPS5, Multichannel systems) at the speed of 1 ml*min^− 1^ with physiological salt solution (PSS) containing [mM] 150 NaCl, 4 KCl, 2 CaCl_2_, 1 MgCl_2_, 5.6 glucose and 25 HEPES (pH 7.4). After recording of the baseline fluorescence, **(i)** we spiked an interval of 10 µM histamine into the perfusion solution, (ii) and delivered sequential high-calcium pulses (5 mM, 10 mM, and 50 mM) with each pulse administered for 10 s at a flow rate of 3 ml/min. Time-lapse images of fluorescence intensity were captured with a Leica DMi8 at one second intervals (1 Hz) using a 20x/1.4 NA lens at 37 °C in 5% CO_2_. The mtCa^2+^ was recorded with a [green, Ex 488 nm | Em 510 nm] and the cytosolic Ca^2+^ was simultaneously recorded with a [red, Ex 555 nm | Em 584 nm] filter setting.

### ΔΨ_M_ measurement in living cells

For the measurement of the mitochondrial membrane potential (MMP, ΔΨ_M_), cells were equilibrated with Tetramethylrhodamine methyl ester (TMRM, 25 nM) and Hoechst 33,347 (50 nM) for 30 min at 37 °C, 5% CO_2_. Images were recorded on Leica DMi8 using a 20x/1.4 NA lens with [red, Ex 555 nm | Em 584 nm] filter settings. CellProfiler v4.2.8 analysis software was used for image analysis [31].

### Determination of the mtDNA copy number

Relative mtDNA copy numbers were determined by qPCR using the amplification curves of a gene encoded on the mtDNA (*MT-ND1*, NC_012920.1) and a single copy gene on the nuclear DNA (*NDUFV1*, NM_001166102.2) using oligonucleotide primers (see Supplemental Table S2) with the SYBR green technology [22] on a TRIO combi cycler (Biometra, 846-2-070-720).

### Statistical analysis

We used Graph Pad Prism v10.4.1 software to run all statistical analyses, which are detailed in the figure captions. Data are presented as mean ± standard deviation (SD), unless specified otherwise. Details on the statistical tests can be found in the corresponding figure legends.

### Experimental methods

Further experimental methods are provided in the Supplemental material section.

## Results

### MCUR1 loss-of-function is associated with proximal muscle weakness and muscle atrophy

**Fig. 1:**
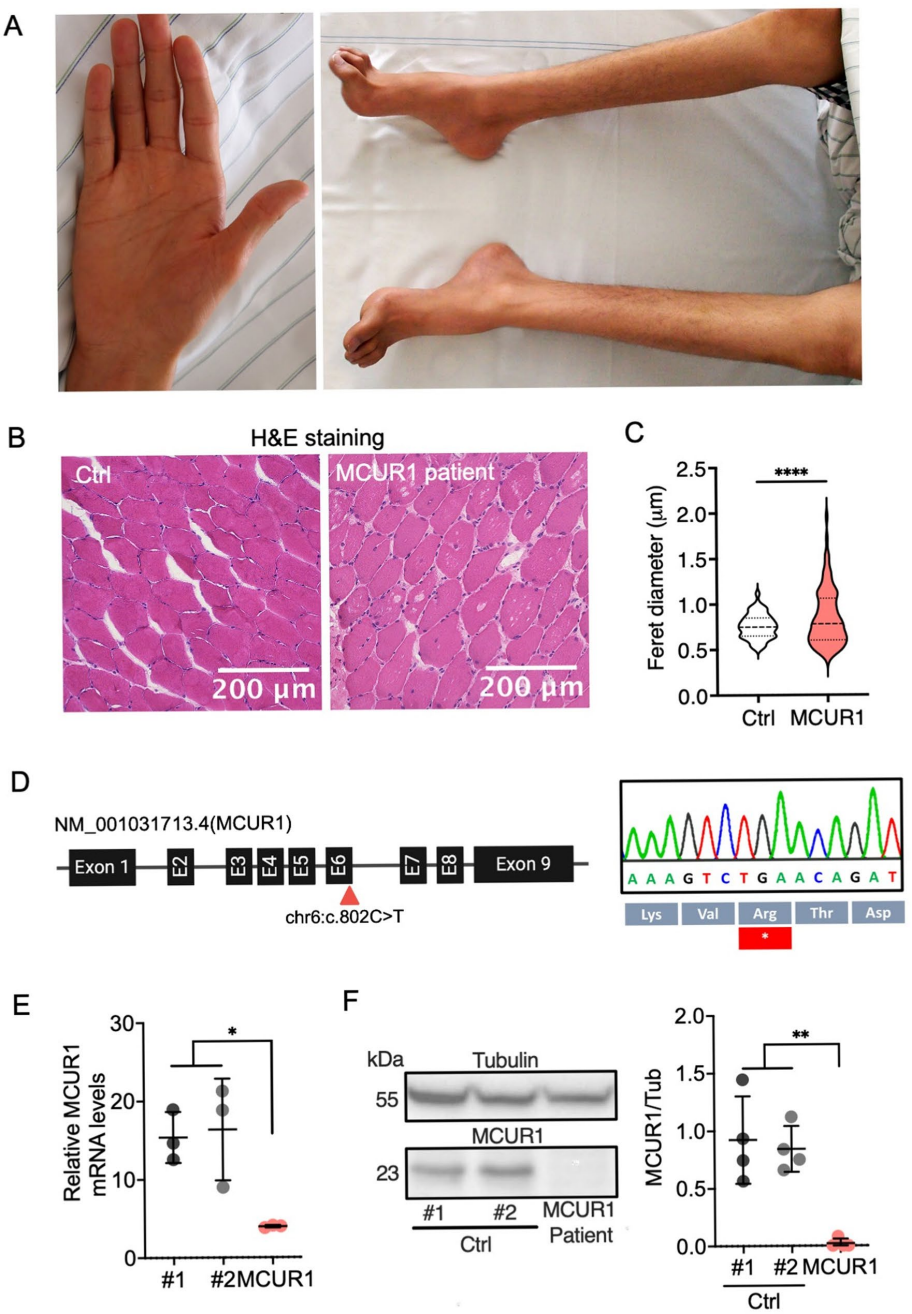
Loss-of-function mutations in *MCUR1* identified in a patient with muscle atrophy. **A** Patient’s hands presenting thenar muscle atrophy and feet with a high arch (cavus foot deformity). **B** Hematoxylin and eosin staining of healthy and patient-derived quadriceps biopsy specimens showing central nuclei and multiple vacuoles inside the muscle fibers. **C** Violin plot showing the distribution of muscle fiber diameters in healthy control and MCUR1-patient muscle. Approximately 200 fibers were analyzed per muscle, with a total of *n =* 6 sections per group. The width of each violin represents data density. The central line indicates the median (0.749 for controls and 0.781 for the MCUR1 patient) with quartiles. Statistical significance was assessed using an unpaired t-test. **D** The *MCUR1* variant in exon 6 was confirmed by Sanger sequencing and annotated on the *MCUR1* transcript (NM_006077.3). **E** Scatter plot of the qPCR analysis demonstrating a significant reduction of *MCUR1* mRNA copies in the MCUR1-deficient patient fibroblasts [*MCUR1* per 1,000 *GAPDH* copies]; error bars represent the mean ± standard deviation (SD) of three biological replicates. **F** Immunoblot showing MCUR1 expression in control fibroblasts and its absence in patient fibroblasts. The molecular weight of the bands is indicated on the left; tubulin was used as a loading control; 100 µg protein were loaded onto each lane. Bands of four biological replicates were analyzed by densitometry and depicted as a scatter plot with error bars representing the mean ± standard deviation (SD). Statistical significance was determined using the Mann-Whitney-U-test in (**E**) and (**F**). *, *p* < 0.05; **, *p* < 0.01; ****, *p* < 0,0001; Ctrl, control

We describe an adolescent patient, son of first degree cousins from Yemen with two healthy sisters, who experienced mild proximal muscle weakness and atrophy in his legs from nine years onward. At 15 years, his muscle weakness had progressed to his lower thighs, feet (progressive cavus foot deformity), and hands (interdigital muscle atrophy, Fig. 1A). Furthermore, he showed a positive Gowers sign. His creatine kinase (CK) levels were constantly elevated to > 3,800 U/L (*N* < 190) suggestive of myofiber damage and fibrosis, which could be confirmed by muscle ultrasound. Serum lactate levels were normal [14.6 mg/dl, *N* < 19], but his pyruvate levels were mildly increased [1.5 mg/dl, *N* < 0.9]. EMG confirmed myopathy with massive spontaneous activity, polyphasic potentials, and reduced motor unit potential amplitudes. Motor and sensor neurography, visual evoked potentials (VEP), EEG, and testing of his intellectual function were all normal. He did not have a cardiomyopathy, but Wolff-Parkinson-White syndrome was successfully treated by ablation at 17 years. The histological investigation of his muscle biopsy specimen has been reported in detail before [25] and revealed an autophagic vacuolar myopathy with variation in fiber size and an increased frequency of accumulated vacuoles (Fig. 1B, C, Supplemental Fig. S1).

The consanguineous family background indicated a likely autosomal recessive inheritance pattern and we delineated autozygous regions with a total of 102.5 Mbp on chromosomes 4, 6, 16, and 18 (Supplemental Fig. S2) with > 8 Mbp stretches of homozygous SNPs using AutozygosityMapper [24]. Analysis with MutationTaster2 [23] identified a homozygous nonsense mutation in *MCUR1* [chr6:13,799,118G > A GRCh37 | c.802 C > T (rs372193345) NM_001031713.4 | p.(R268*)] (Fig. 1D). In his family, only the affected patient was carrying this mutation homozygously (Supplemental Fig. S3). Disease-causing mutations on the X-chromosome (e.g., *LAMP2* and *VMA21*) were excluded by segregation analysis and by minor allele frequencies (Supplemental Table S1).

In the gnomAD v2.1.1 database this *MCUR1* variant is listed with a minor allele frequency of 0.00005351 and never in homozygous state [https://gnomad.broadinstitute.org/variant/6-13799118-G-A?%20dataset=gnomad_r2_1, accessed on April 10, 2026]. To assess the physiological relevance of the *MCUR1* mutation, we investigated dermal fibroblasts and muscle tissue from the patient and from matched controls. *MCUR1* mRNA copy numbers were reduced in patient-derived fibroblasts as were the protein levels on Western blot (Figs. 1E, F).

### MCUR1 deficiency reduces mtCa^2+^ influx without disrupting MCU complex assembly and localization

**Fig. 2:**
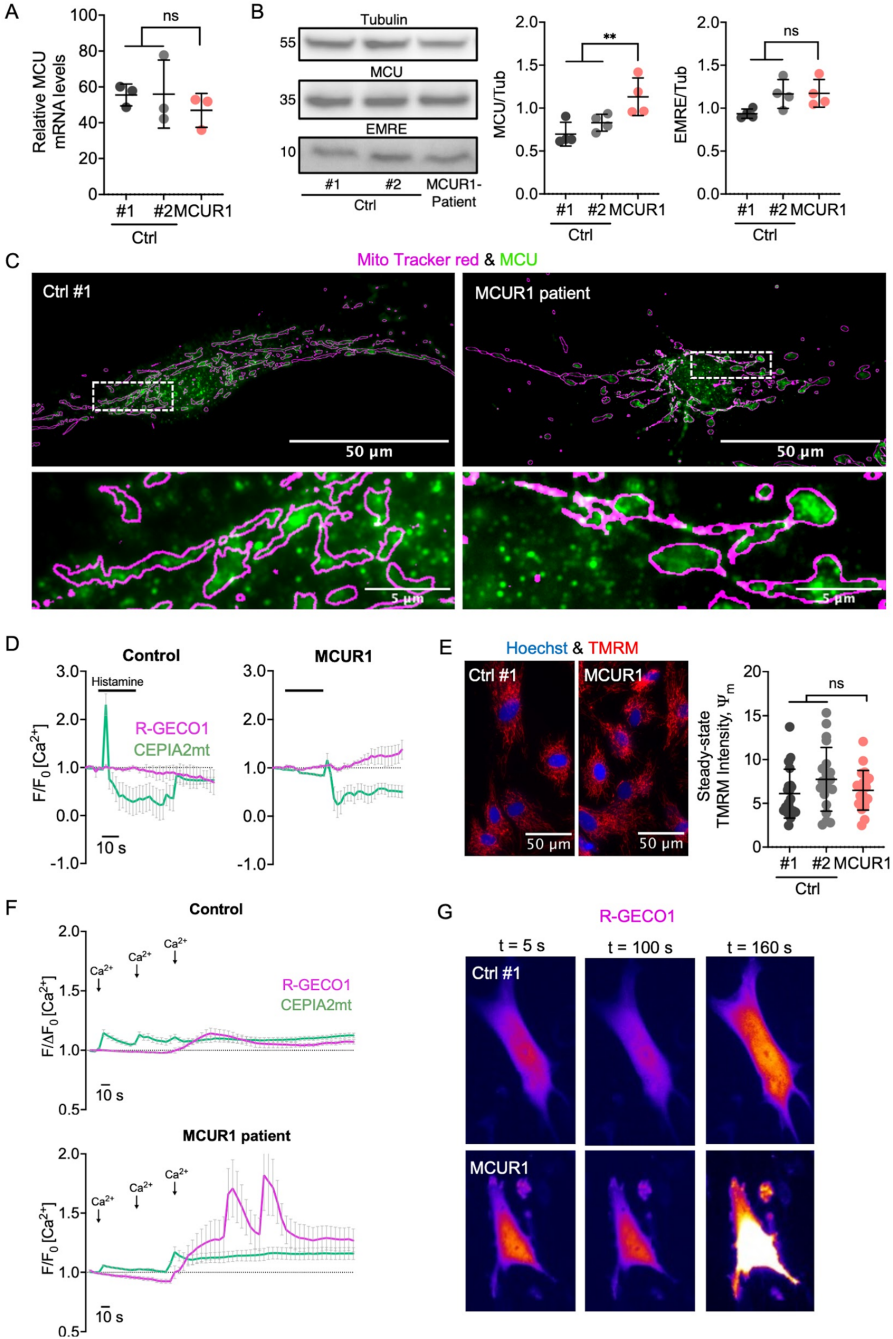
Expression analysis of MCU complex subunits and measurement of mtCa^2+^ influx. **A** qPCR analysis demonstrating MCU mRNA copy numbers in the MCUR1-deficient patient fibroblasts and controls, error bars represent the mean ± standard deviation (SD) of three biological replicates [*MCU* per 1,000 *GAPDH* copies]. **B** Immunoblot showing MCU and EMRE protein expression in control and patient fibroblasts. Protein molecular size is indicated on the left; tubulin is used as a loading control; 60 µg protein were loaded onto each lane; error bars represent the mean ± standard deviation (SD) of four biological replicates. **C** Immunofluorescence microscopy images showing MCU *puncta* formation (green) in single cells. No difference between patient and controls can be seen. The magenta outlines depict the mitochondrial shapes as generated by staining with MitoTracker™ Red FM. **D** Histamine (10 µM) induced cytosolic (magenta) and mtCa^2+^ signals (green) were measured using R-GECO1 and CEPIA2mt reporter constructs in primary fibroblasts from the patient compared to controls. Traces show the F/F_0_ ratio of fluorescence in single cells. F_0_ depicts the baseline fluorescence before stimulation. Traces of Ca^2+^ concentrations are shown as mean ± SD from > 10 traces of four separate experiments. **E** Primary fibroblasts of the patient and from age-matched healthy individuals were stained with TMRM to quantify the ΔΨ_M_. Cells were identified by Hoechst 33,347 staining of their nuclei. *n* = 20 single cells were analyzed in three independent experiments. Scatter blots in A, B, and E show mean ± SD from at least three separate experiments. **F** Ca^2+^ traces recorded from mitochondrial (green) and cytosolic (magenta) compartments in control (top) and MCUR1 patient (bottom) fibroblasts. Relative fluorescence intensity is shown as F/F₀ over time. Sequential extracellular Ca^2+^ pulses (5 mM, 10 mM, 50 mM) are indicated by arrows. Solid lines represent mean responses across biological replicates, with error bars indicating ± SD. **G** Image representation of single cells showing and increase in R-GECO1 fluorescence after subsequently increasing extracellular Ca^2+^ pulses. For pairwise comparisons the Mann-Whitney-U-test was used: **, *p* < 0.01; ns, not significant; Ctrl, control

As MCUR1 has been reported to directly bind to MCU and EMRE to form the active MCU complex (Supplemental Fig. S4A) [28], we evaluated the mRNA transcription for subunits of the MCU complex in patient muscle and fibroblasts. RNA sequencing did not detect any deviating mRNA levels for the other MCU-complex subunits except for *MCUR1* mRNA (Supplemental Fig. S4B, C). The findings for *MCU* gene expression were confirmed by RT-qPCR (Fig. 2A). Moreover, the absence of MCUR1 did not affect the synthesis of MCU or EMRE proteins in patient cells (Fig. 2B) nor the subcellular location of the MCU *puncta* at the inner mitochondrial membrane (Fig. 2C).

To evaluate the functional consequences of MCUR1 deficiency, we analyzed Ca^2+^ homeostasis in fibroblasts from our patient and from healthy individuals. Using intraorganellar fluorescent reporter constructs (R-GECO1 and CEPIA2mt [26]), we measured fluorescence after external stimulation with histamine (10 µM). Histamine-induced mtCa^2+^ uptake of mitochondria was diminished in MCUR1-deficient fibroblasts, while there was a relative increase of the cytosolic Ca^2+^ concentration (Fig. 2D) indicating a malfunction of mitochondrial calcium clearing from the cytoplasm after its release from the endoplasmic reticulum (ER). To validate these results, we additionally analyzed calcium homeostasis in cells challenged with pulses of increasing (5 to 50 mM) extracellular Ca^2+^ concentrations. Again, patient fibroblasts displayed an elevation of cytosolic calcium concentrations (Figs. 2F, G). Furthermore, mtCa^2+^ uptake was significantly reduced and almost absent during the first two calcium pulses (Fig. 2F).

The major driving force behind the mtCa^2+^ uptake is the strongly negative potential across the inner mitochondrial membrane (ΔΨ_M_), which is generated and maintained by the mitochondrial electron transport chain. To investigate whether the ΔΨ_M_ would be reduced in patient fibroblast mitochondria (as an alternative explanation for the reduced mtCa^2+^ influx), we evaluated the ΔΨ_M_ using the potentiometric fluorescent Tetramethylrhodamine methyl ester (TMRM) dye. However, the *MCUR1* nonsense mutation did not alter ΔΨ_M_, suggesting that MCUR1 mainly regulates mtCa^2+^ conductance across the inner mitochondrial membrane and not the ΔΨ_M_ (Fig. 2E).

### MCUR1 deficiency reduces ATP production and oxygen consumption rate (OCR)

**Fig. 3:**
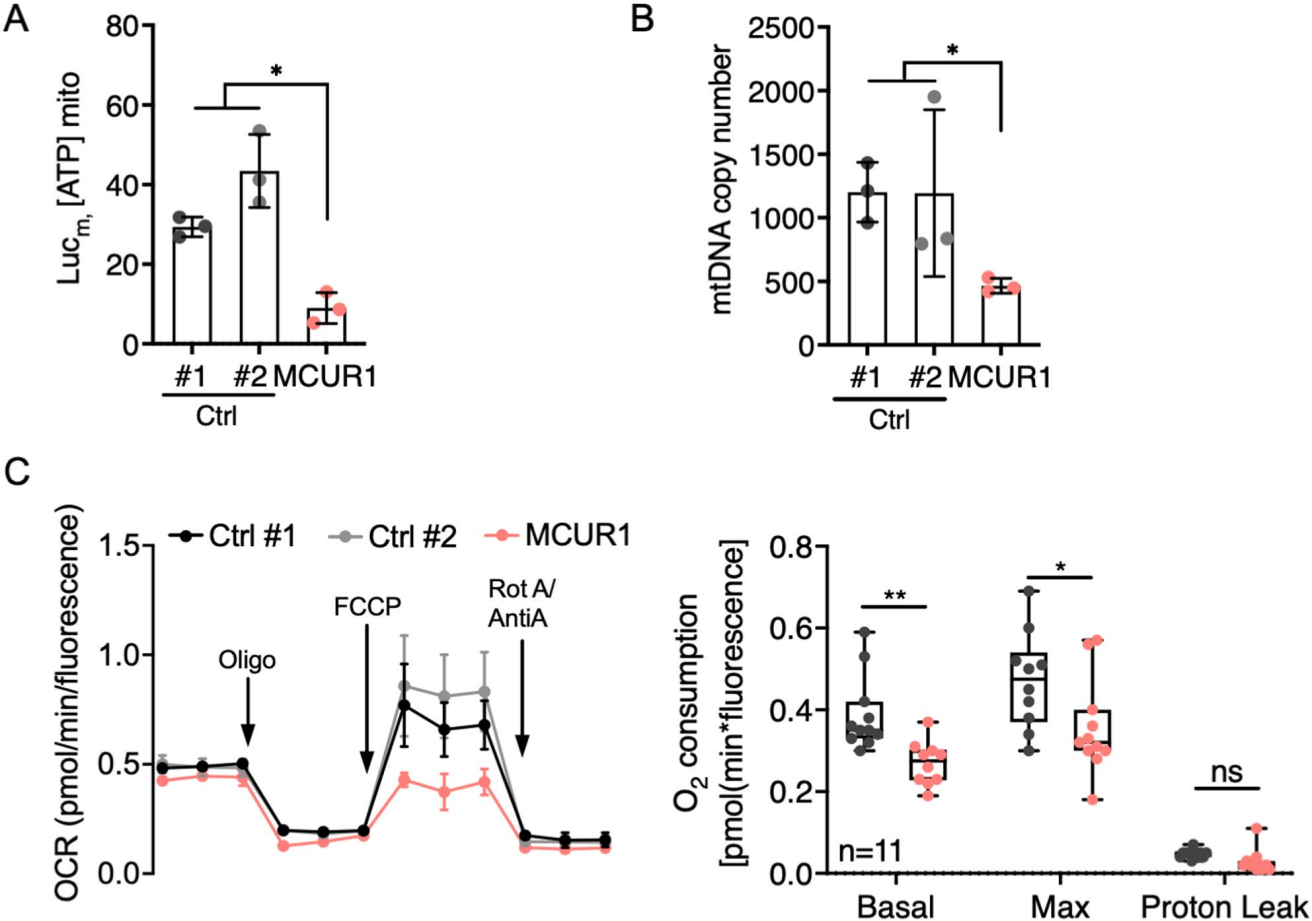
Effect of MCUR1 deficiency on mitochondrial oxygen consumption and mitochondrial energy metabolism. **A** Scatterplot of mitochondrial ATP production in control and MCUR1-patient fibroblasts measured by an ATP-luminescence assay by recording relative fluorescent units (Luc_m_). **B** qPCR analyses for mtDNA copy numbers were performed in controls and MCUR1-patient cells. Scatter plot shows the mean ± SD from three separate experiments. **C** Normalized oxygen consumption rates (OCR) of intact control and patient fibroblasts were measured using a Seahorse™ flux analyzer. Basal respiration, maximal respiration (in the presence of 1 µM FCCP), and leakage respiration (in the presence of 2.5 µM oligomycin) are depicted. Graphs show mean ± SD of at least three separate experiments. Pairwise comparisons in **A** and **B** were done by the Mann-Whitney-U-test and in **C** by the t-test: *, *p* < 0.05; **, *p* < 0.01; ns, not significant; Ctrl, Control

During muscle contraction, Ca^2+^ is released from the sarcoplasmic reticulum into the sarcoplasm, which is then partly taken up by the mitochondria and partly directly moved back into the SR by the SERCA pumps. The mtCa^2+^ influx regulates the activities of the pyruvate dehydrogenase complex (PDHc), the tricarboxylic acid (TCA) cycle dehydrogenases, the OXPHOS system, and the F_1_F_O_-ATPase, thereby closely coupling ATP demand during muscle contraction to ATP production [7, 13, 18, 21]. To assess the effect of MCUR1 deficiency on the ATP production, we used a mitochondrial matrix targeted luciferase that reports mitochondrial matrix ATP concentrations in living cells *via* light emission. We found reduced ATP production in the patient fibroblasts (Fig. 3A). In contrast, the activities of isolated OXPHOS and Pyruvate dehydrogenase (PDH) complexes from the patient’s fibroblasts were within the normal range (Supplemental Fig. S5A). In addition, we performed histochemical analyses of NADH-TR, succinate dehydrogenase (SDH), and cytochrome C oxidase (COX) and did not observe any differences compared to controls (Supplemental Fig. S5B).

Given the central role of mitochondria in energy homeostasis, we investigated whether MCUR1 deficiency affected mitochondrial biogenesis and degradation. Quantification of the mtDNA copy number revealed a significant reduction in the patient-derived fibroblasts, suggesting either impaired mitochondrial biogenesis, reduced mtDNA replication, increased degradation, or a combination thereof (Fig. 3B).

Although isolated OXPHOS complexes displayed normal enzymatic activities (Supplemental Fig. S5A), we assessed the mitochondrial respiration in intact coupled cells using Seahorse™ extracellular flux analysis to assess OXPHOS regulation within the cellular context. MCUR1-deficient fibroblasts exhibited reduced basal oxygen consumption rates (OCR) and a lower maximal respiratory capacity, whereas the proton leak was not affected (Fig. 3C).

Given the reduced mtCa^2+^ influx, ATP production, and OCR in MCUR1-deficient cells, we investigated whether Pyruvate dehydrogenase complex (PDHc) activity might be altered [12]. The PDHc can be inactivated by the PDH kinase (PDK) through phosphorylation of three serine residues of the E1-subunit, while activation is achieved by the phospho-PDH phosphatase (PDP1), which is dependent on the mtCa^2+^ concentration. PDHc activity was measured by incubating the patient fibroblasts with ^13^C-labeled substrates. The ratio of the secretion of ^13^C₄-citrate and of ^13^C₃-α-ketoglutarate by the carnitine acetyltransferase (CAT) pathway versus the PDH enzyme complex (CAT/PDH ratio) provides a measure for the overall PDHc activity [14]. Substrate concentrations were measured by ^13^C-NMR-spectroscopy [30]. All PDHc activity measurements were within the normal range (Supplemental Fig. S5A).

### MCUR1 deficiency promotes autophagy pathways in muscle

**Fig. 4:**
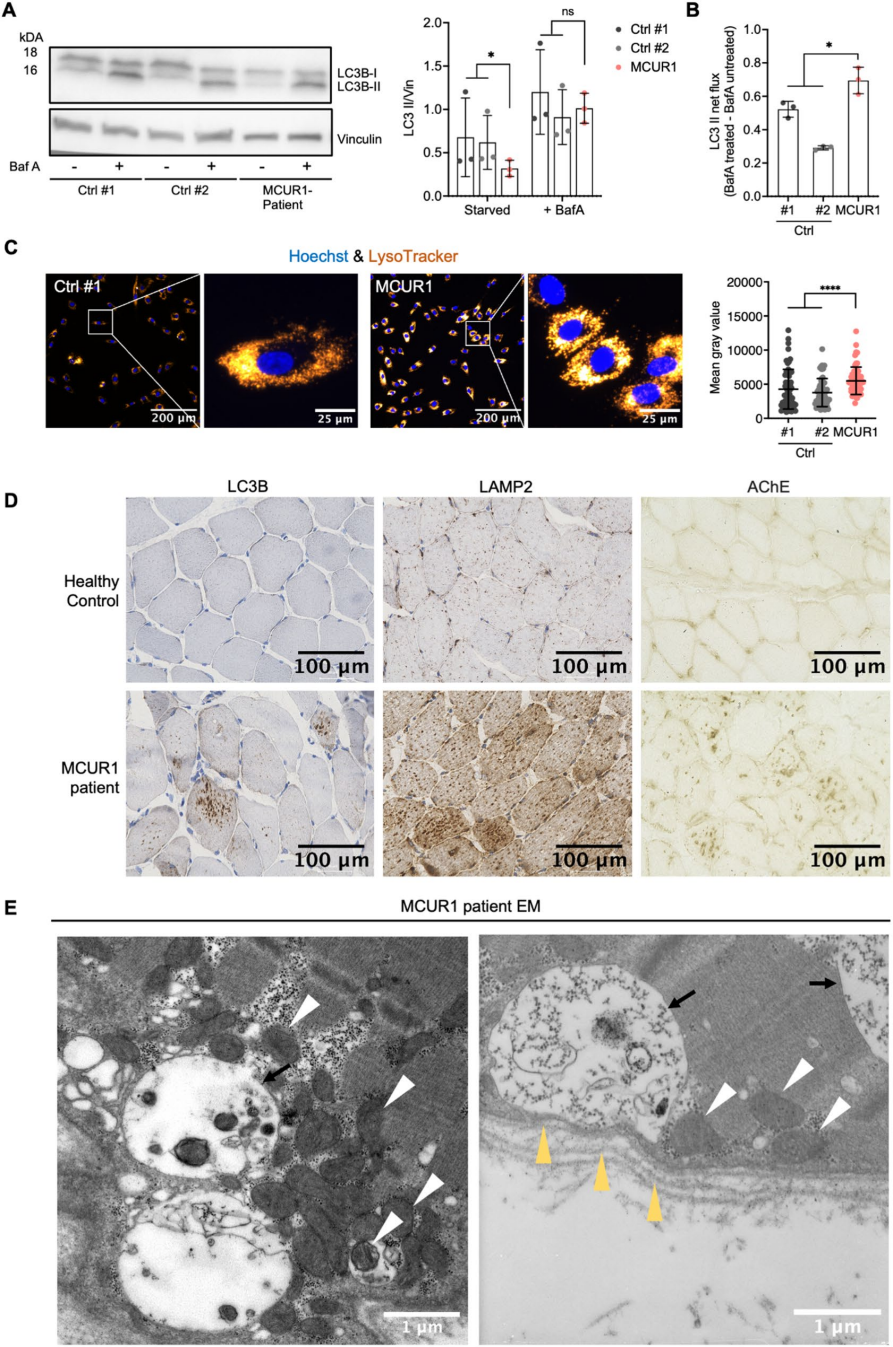
Protein expression of autophagic markers; histologic investigations. **A** Western blot analysis of LC3B-I and LC3B-II in patient and control fibroblasts after starvation in serum free media in the absence or presence of the lysosomal fusion inhibitor bafilomycin A1 (BafA1) (*n* = 3 technical replicates for each cell line). **B** LC3B-II net flux was evaluated in patient and control cells. **C** Counterstain with LysoTracker™ and Hoechst 33,342 of patient fibroblasts versus controls (*n* = 3) show increased lysosomal acidification in patient cells. **D** Histochemistry of skeletal muscles for acetylcholine esterase activity (AChE), LAMP2, and LC3B shows increased staining for all 3 proteins in the patient’s muscle sample. **E** Electron microscopy of skeletal muscle from an MCUR1 patient depicts large autophagic vacuoles, partially localized in the subsarcolemmal region. Vacuoles are surrounded by a single membrane and lack a basement membrane (black arrows). Mitochondria in close proximity to or within the vacuoles are indicated by white arrowheads. Sarcolemmal membrane and basement membrane are marked by yellow arrowheads. Statistical significances in **A**–**C** were determined using the Mann-Whitney-U-test: *, *p* < 0.05; ****, *p* < 0.0001; ns, not significant; Ctrl, Control; EM, electron microscopy

One potential adaptive response to mitochondrial dysfunction is autophagy [20]. To investigate whether autophagic activity was affected by MCUR1 loss-of-function, we measured LC3B-II accumulation under stress by immunoblotting. Patient and control fibroblasts displayed LC3B-II accumulation upon starvation and lysosomal inhibition with bafilomycin A1 (BafA1) (Fig. 4A). However, LC3B-II net flux was elevated in the patient cells, which was calculated by the difference of intensities for LC3B-II staining of BafA1-­treated minus BafA1-­untreated samples (Fig. 4B). BafA1 inhibits the v-ATPase, preventing lysosomal acidification and autophagosome-lysosome fusion, therefore LC3B-II should accumulate if autophagosomes are being formed normally. Interestingly, LC3B-II basal levels during starvation were already lower in patient-derived cells without BafA1-treatment, hinting towards increased baseline autophagic activity (Fig. 4A, B).

To evaluate lysosomal function, we stained fibroblasts with LysoTracker™ Red DND-99, a dye that can be used to evaluate alterations of the lysosomal pH in living cells. We found increased fluorescence in patient fibroblasts, indicating a lower pH and elevated lysosomal activity (Fig. 4C).

The results prompted us to shift our focus to muscle, as the primarily affected tissue. Histology revealed large vacuolar structures, suggesting accumulation of autophagic material. The membranes of these vacuoles were positive for acetylcholine esterase (AChE) activity. In addition, staining for the autophagy-related proteins LAMP2 and LC3B showed abnormally increased aggregations in the muscle fibers (Fig. 4D). Electron microscopy of the patient’s quadriceps muscle revealed large vacuoles partially with subsarcolemmal localization. The vacuoles were delimited by a single membrane, did not contain any basement membrane, and were hence termed ‘*sarcolemma-like membranes*’ (Fig. 4E, *right panel*). In some vacuoles we find the remains of mitochondria as a correlate of mitophagy (Fig. 4E, *left panel*).

**Fig. 5:**
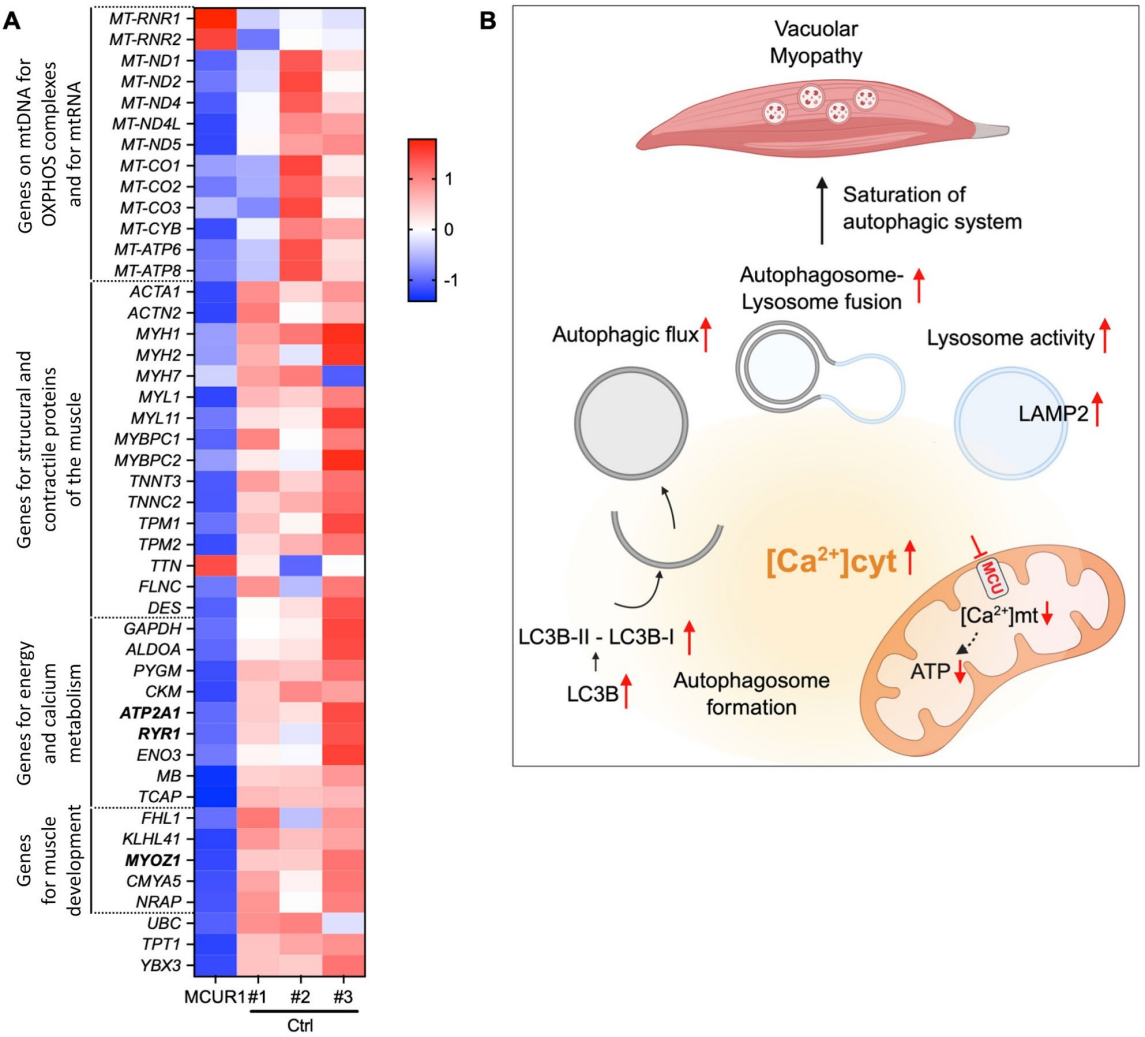
RNA-seq analysis of muscle samples. **A** Visualization of RNA-seq results with a heatmap suggesting transcriptional changes due to MCUR1 deficiency of muscle tissue from three healthy controls and the MCUR1-patient. Differentially expressed genes (DEGs) are represented with log2 fold change (x) of |x| ≥ 1 and a false discovery rate (FDR) of < 0.05. **B** Schematic representation of our hypothesis about MCU regulation of autophagy. Impaired mtCa^2+^ uptake due to the *MCUR1* mutation leads to reduced mtCa^2+^ influx thereby accumulating cytosolic Ca^2+^ and decreasing mitochondrial ATP production. This enhances autophagy with increased LC3B levels in muscle cells and an increased autophagic flux (autophagosome production). At the same time, lysosome activity is upregulated leading to an increase in autophagosome-lysosome fusion. The ensuing disequilibrium between autophagic sequestration and completion of the degradative process (= autophagic stress [4]) produces deposition of vacuolar material in the muscle tissue

In order to get a wider perspective of the MCUR1-dependent transcriptional changes, we performed bulk RNA sequencing on muscle samples of three healthy controls *versus* the MCUR1-deficient patient. We found 1992 differentially expressed genes (DEGs), among them, 776 up-regulated and 1216 down-regulated (FDR < 0.05; log2 fold change (x) of |x| ≥ 1). MCUR1 deficiency had caused a clear reduction of the steady state levels of multiple mRNAs that are transcribed from the mtDNA and code for most of the mtDNA encoded subunits of the OXPHOS complexes I, III, IV and V. This is in agreement with the reduced mtDNA copy number and could explain the overall reduced OCR and ATP-production. Interestingly, only the mitochondrial ribosomal RNA was highly upregulated, probably as a compensatory measure of the MCUR1-deficient cells. The mRNA of key calcium-handling genes (*RYR1*,* ATP2A1*) and metabolic enzymes (*CKM*,* PYGM*) showed reduced mRNA expression. Additionally, we found the broad downregulation genes encoding structural and contractile proteins of the muscle (Fig. 5A).

As we saw a general reduction of LC3B-II levels in patient cells, we investigated whether the mRNA expression of autophagy-related genes (*LC3B2*, *LAMP1*, *LAMP2*, *PINK1*, *P62*, and *VMA21*) was altered, but did not find any clear deviations from the normal range (Supplemental Fig. S6).

## Discussion

In this study, we present data on the physiological effects of a biallelic *MCUR1* mutation. The patient’s leading symptoms comprise early-onset proximal muscle weakness, generalized muscle atrophy, and cavus foot deformity. We identified a homozygous nonsense variant in *MCUR1* that is associated with vacuolar myopathy. Mutations in the coding regions of genes on the X-chromosome that are known to be associated with vacuolar myopathy (*LAMP2* and *VMA21*) were excluded (Supplemental Table S1).

Despite its known role in the MCU complex, we observed that MCUR1 deficiency did not disrupt the assembly or localization of the MCU complex. Expression levels of the channel proteins MCU and EMRE and of other regulatory subunits (MICU1, MICU2, and MICU3) were unaltered. However, MCUR1 deficiency reduced mtCa^2+^ uptake and increased cytosolic Ca^2+^ accumulation upon histamine stimulation or by extracellular calcium challenge, suggesting a regulatory rather than structural role for MCUR1. Notably, these defects occurred independently of changes in Ψ_m_, supporting the idea that MCUR1 primarily regulates mtCa^2+^ influx. This aligns with prior reports indicating that MCUR1 functions as a critical modulator of mtCa^2+^ influx rather than a core component of the uniporter complex [17, 28, 29].

In patient fibroblasts, we observed a reduction of mitochondrial ATP production and of basal oxygen consumption despite preserved enzymatic activities of the isolated PDH and OXPHOS complexes. This dissociation suggests that defective ATP production arises rather from impaired regulation of cytosolic Ca^2+^ transients than from intrinsic dysfunction of isolated complexes of the respiratory machinery. The observed reduction in mtDNA copy numbers may further aggravate this metabolic phenotype. Whether this is a direct consequence of impaired Ca^2+^ signaling or a secondary adaptation to chronic OXPHOS dysfunction remains unsolved. Our findings align with previous studies showing that MCU or MCUR1 regulate oxidative metabolism in skeletal muscle, mitochondrial ATP output, and overall muscle function [11, 12, 18].

Loss of MCUR1 or MCU activity has also been linked to reduced muscle mass and strength in mouse models, *and MCUR1* was found to be downregulated on the mRNA level in older sarcopenic patients, suggesting that both proteins have a role in muscle regeneration [17, 28, 29]. Mammucari *et al.* show that AAV9-mediated shRNA silencing of MCU in skeletal muscle of mice results in severe muscle atrophy accompanied by an increase of intracellular vacuoles [18]. Furthermore, *MCUR1* knock-down in HeLa cells led to suppressed mtCa^2+^ influx, reduced OXPHOS and lower ATP levels [17, 28]. In these studies, HeLa cells were transduced with adenoviruses carrying short hairpin RNAs (shRNA) designed to reduce the expression of several MCU subunits. Finally, *MCUR1 *knock-down did result in elevated LC3 puncta formation and the ultrastructural analysis of c*MCUR1* KO cardiomyocytes displayed a higher number of autophagosomes [6, 28].

The presence of numerous autophagic vacuoles with sarcolemmal features (AVSF) in the muscle of our patient, a feature seen in autophagic vacuolar myopathies such as Danon disease and X-linked myopathy with excessive autophagy (XMEA), hints towards a defect in autophagy. Autophagy is tightly regulated by cytosolic Ca^2+^ and its elevation may either promote or inhibit different stages of the process [10]. While the molecular defects underlying these disorders differ, the shared presence of AVSFs underscores the vulnerability of skeletal muscle to imbalanced autophagy-lysosome coupling. In our patient, we see both an increase in LC3B levels in many muscle fibers and, at the same time, an elevation of LAMP2 staining. In line with this, we found increased LysoTracker™ staining, suggesting that lysosomal acidification and their degradative capacity are preserved or even elevated. The increased autophagic flux in starved fibroblasts after bafilomycin A1 treatment lets us hypothesize that increased cytosolic Ca²⁺ concentrations either impede autophagosome-lysosome fusion or that the elevated autophagic flux causes “autophagic stress” with incomplete autophagosome degradation and their deposition in the muscle tissue. The concept of “autophagic stress” implies an imbalance between the rates of autophagic sequestration and completion of the degradative process [4].

The rapid clearance of Ca^2+^ from the cytosol is achieved by both the SERCA pumps of the SR and by mtCa^2+^ uptake via the MCU. The involvement of Ca^2+^ signaling in the regulation of autophagic activity has been extensively investigated, however, several mechanistic aspects remain unresolved. Elevations in cytosolic Ca^2+^ have shown to either stimulate or suppress autophagy [2, 15]. Further experimental work is needed to determine the detailed mechanism.

In summary, MCUR1 deficiency leads to impaired mtCa^2+^ uptake without disrupting uniporter assembly, resulting in reduced mitochondrial performance, ATP depletion, and ultimately dysregulated autophagy. However, the interpretation of these findings is limited by the analysis of a single patient. As a consequence, inter-individual variability of MCUR1 deficiency could not be evaluated. Moreover, because the clinical phenotype is restricted to skeletal muscle, the use of dermal fibroblasts represents an inherent limitation and may underestimate the severity of tissue-specific effects. Consequently, some aspects of MCUR1 deficiency observed in fibroblasts may underestimate the true impact of the variant in the primarily affected tissue. This restricts our ability to generalize the results beyond this single individual. A more complete understanding will therefore require the inclusion of additional patient samples and complementary studies in disease-relevant cell types.

## Supplementary Information

Below is the link to the electronic supplementary material.

Supplementary Material 1

Supplementary Material 2

## Data Availability

Raw data were generated at Charité—Universitätsmedizin Berlin, Germany. The RNAseq dataset generated in this study is available from the corresponding author upon reasonable request. The patient and his family members’ whole exome datasets are not publicly available due to genetic data privacy regulations. The novel variant was submitted to the ClinVar database (https://www.ncbi.nlm.nih.gov/clinvar;%20accession%20number%20SCV006307813).
