# Supplementary material for: Biallelic MCUR1 nonsense mutation associated with vacuolar myopathy and altered mitochondrial calcium signaling
Source: Acta Neuropathol Commun. 2026 May 5;14:107. doi: 10.1186/s40478-026-02313-y (PMC13162457; doi:10.1186/s40478-026-02313-y)
Supplement: Supplementary file 1 — Supplementary Material 1 [file 40478_2026_2313_MOESM1_ESM.pdf]

Supplemental materials

Biallelic *MCUR1* nonsense mutation associated with vacuolar myopathy and altered mitochondrial calcium signaling

Anna Maria Haschke, Anja von Renesse, Eugenio Graceffo, Susanne Morales-Gonzalez, Alessandro Prigione, Christoph Hübner, Werner Stenzel, and Markus Schuelke

Supplemental Figures

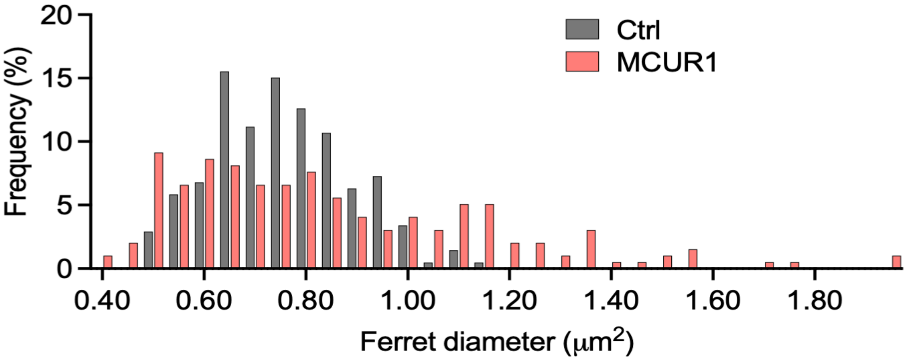

Supplemental Figure S1. Mean fiber size and size dispersion of healthy control and MCUR1-patient muscle (>200 fibers per muscle; n=6 sections analyzed); the patient's muscle shows a wider dispersion of muscle fiber sizes with a slight shift of the mean fiber diameter to the left (Control median = 0.749; MCUR1 patient median = 0.781).

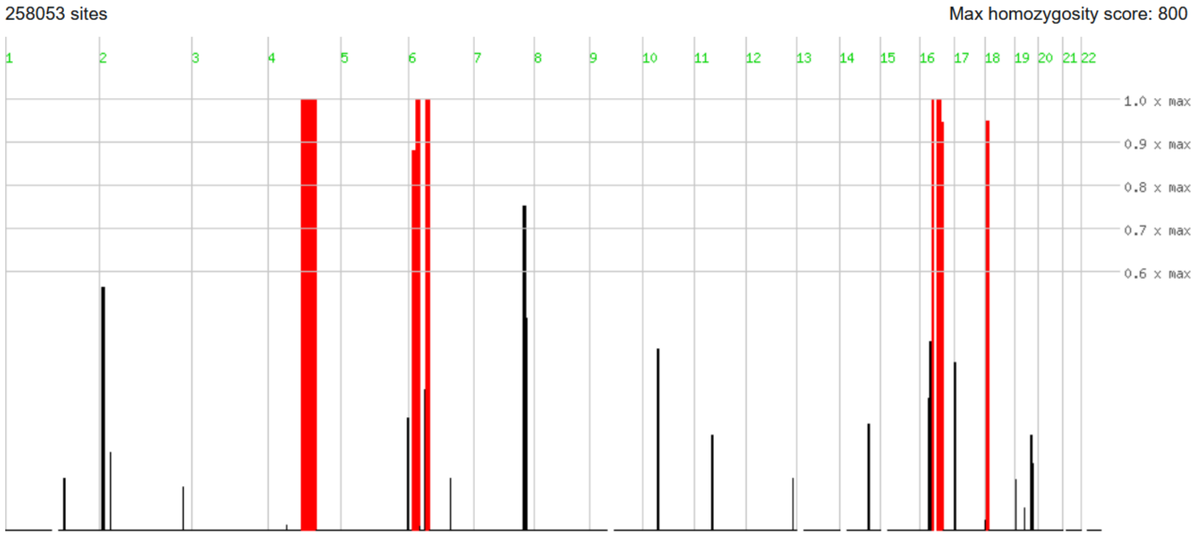

Supplemental Figure S2. Regions that are autozygous over >8 Mbp in the exome of the index patient in contrast to the exomes of the two healthy sisters and both healthy parents as delineated by the autozygositymapper

software [7]. The entire autozygous region stretches over 102.5 Mbp. The *MCUR1* gene is located at chr6:13,786,781-13,814,792 (GRCh37).

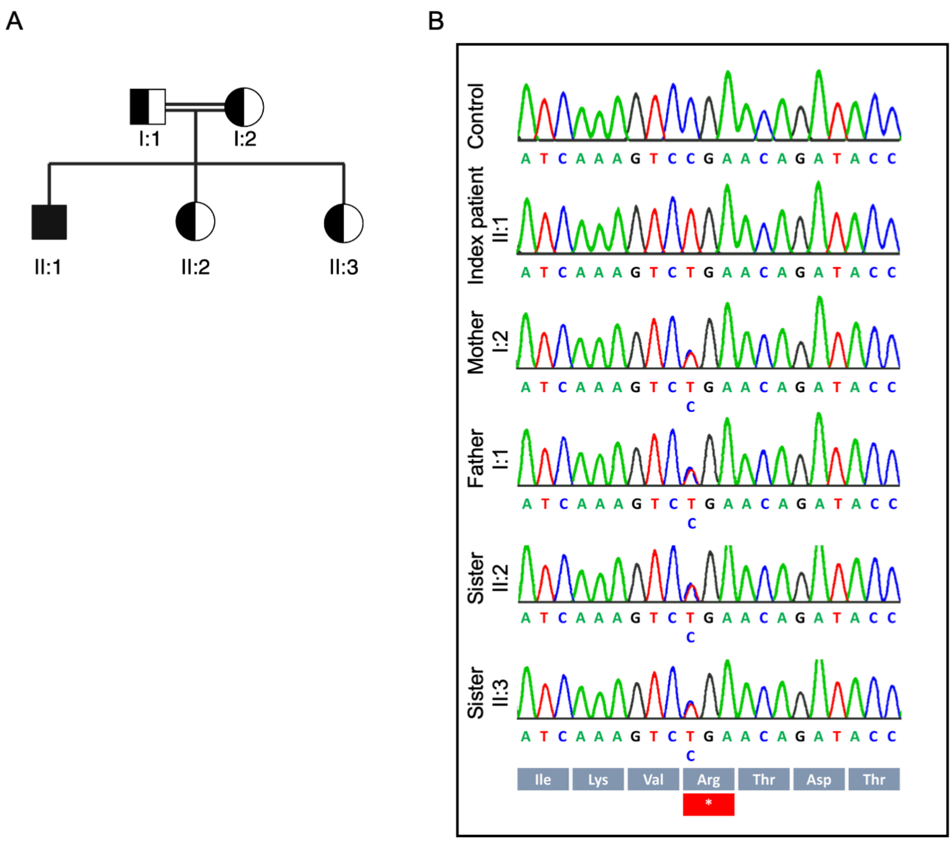

Supplemental Figure S3. (A) Pedigree of the consanguineous family with the index patient (II:1, filled symbol). **(B)** Segregation of the *MCUR1* variant [c.802C>T, NM_001031713.4 | p.(R268*)] in the patient's family.

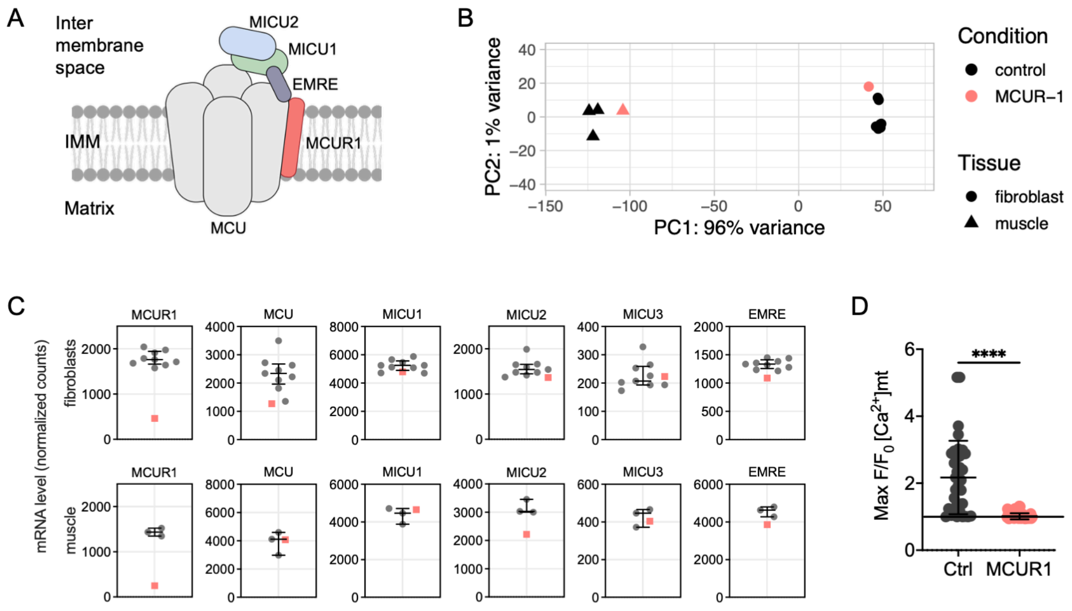

Supplemental Figure S4. (A) Schematic overview of the composition of the MCU complex and its protein subunits. **(B)** Principal component analysis (PCA) of the transcriptome clearly distinguishes between fibroblasts (patient and n=3 controls) and muscle samples (patient and n=3 controls), but also between the MCUR1-patient and the controls. **(C)** mRNA expression levels (normalized counts are given as TPM values) of genes encoding subunits of the MCU complex measured by RNA sequencing of fibroblasts (n=9 age-matched controls *versus* the patient) and human muscle biopsy samples (n=3 age-matched controls versus the patient). **(D)** Histamine(10 µM) induced mtCa^{2+} signals from the affected individual compared to controls. The plot demonstrates the peak increase of the mtCa^{2+} signal against the signal during basic steady state. Peak relative mtCa^{2+} concentrations are shown as mean ± SD from four separate experiments. Statistical significance was assessed using an unpaired t-test, ****, p < 0,0001.

A

Enzyme activity	Control range	Patient activity	Units
Complex I	279 - 1076	879 | 766	mU/U COX
Complex II	375 - 2692	1099 | 1072	mU/U COX
Complex III	632 - 3534	2479 | 1175	mU/U COX
Complex II + III	325 - 649	765 | 622	mU/U COX
Complex IV	288 - 954	354 | 429	mU/U CS
Complex V	480 - 2705	1837 | 1450	mU/U COX
Citrate synthase	151 - 449	399 | 387	mU/U protein
Pyruvate dehydrogenase complex (PDHc)	9.7 - 36	17.7	mU/U CS
Ratio $^{13}C_4$-citrate secretion (CAT/PDH)	0.97 -1.81	1.70	
Ratio $^{13}C_3$-α-ketoglutarate secretion (CAT/PDH)	1.03 - 1.59	1.45	

B

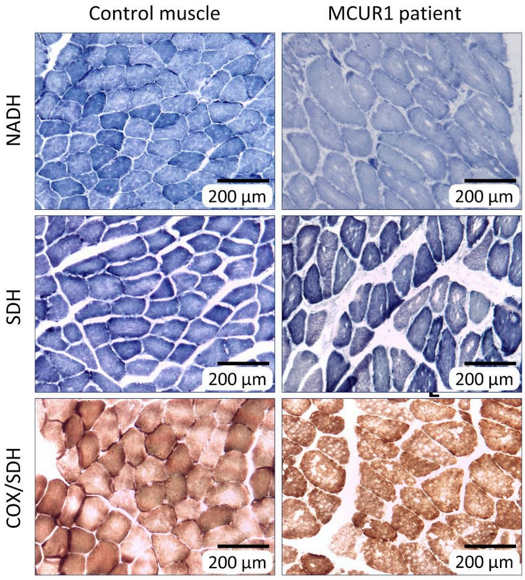

Supplemental Figure S5. (A) Measurements of the activities of the isolated respiratory chain complexes and the PDHc in cultured skin fibroblasts of the patient in comparison to reference values. CAT, carnitine acetyltransferase; COX, cytochrome C oxidase; CS, citrate synthase; PDH, pyruvate dehydrogenase. The OXPHOS complexes were measured in two independent biological replicates of skin fibroblasts. Replicate measurements are separated by a vertical line. **(B)** Mitochondria specific histologic stainings of NADH (complex I), SDH (succinate dehydrogenase, Complex II), and COX/SDH (complex IV + Complex II) show normal staining patterns, the patient's muscle shows multiple vacuolar deposits inside the muscle fibers.

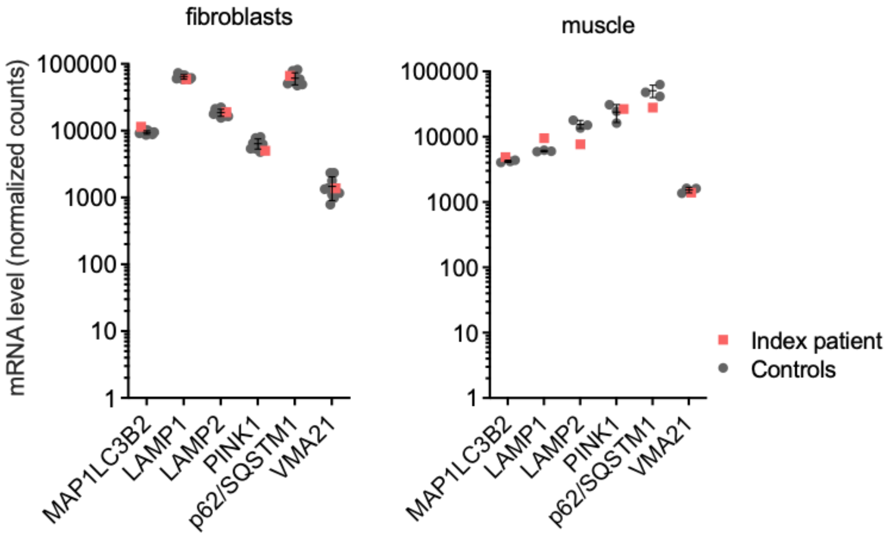

Supplemental Figure S6. mRNA expression (normalized counts are given as TPM values) of genes related to autophagy or to those mutated in autophagic vacuolar myopathy as determined by RNA sequencing of fibroblasts (n=9 age-matched controls versus the patient), and of human muscle biopsy samples (n=3 age-matched controls versus the patient). Graphs show mean ± SD.

Supplemental Methods

Fibroblast cell culture

Fibroblasts were derived from skin punch biopsy samples and cultured in DMEM (Gibco, #41966-029) supplemented with 15% foetal bovine serum (Gibco, #10500-064) and 1% penicillin–streptomycin (Gibco, #15140122), and incubated at 37°C in 5% CO_2. The fibroblast line was confirmed to harbor the correct homozygous *MCUR1* mutation by Sanger sequencing prior to further functional studies. Cell lines were routinely tested for mycoplasma contamination.

DNA analysis

Whole exome sequencing was performed using a blood DNA sample of the index patient. The coding exons and flanking intronic regions were enriched using the Agilent SureSelect XT Human All Exon V5 Exome Kit and sequenced as 100 bp paired-end fragments on an Illumina HiSeq2500 machine. The 65 million resulting sequence fragments (FASTQ files) were aligned to the human reference genome (GRCh37.p11, hg19/Ensembl 72) using the BWA-MEM v0.7.8 software. Deviations from the reference sequence were detected and recorded using the GATK v3.1.1 software package and exported as a VCF-file. Each of these variants was then analysed using the MutationTaster2 [6] variant assessment software to identify potentially pathogenic variants. Segregation analysis of the variants in the family was performed by Sanger sequencing. Oligonucleotide primers (see **Supplemental Table S2**) were used to amplify exon 6 of *MCUR1* and sequencing was performed with the dideoxy chain termination method (BigDye Terminator Version 3.1, Thermo Fisher, #4337457) on an ABI 3730 DNA sequencer.

RNA analysis

For RNA-sequencing, total RNA was isolated from patient and control muscle biopsy samples that had been obtained from the quadriceps muscle and from cultured skin fibroblast specimens using using the TRIzol protocol (Invitrogen, #15596026). 500 ng of RNA from each sample were sent for bulk RNA sequencing to the Beijing Genomics Institute on their DNBseq-500 platform. The library preparation included an mRNA enrichment step using oligo(dT)-attached magnetic beads. The strand-specific cDNA library generated 60 million 100 bp paired-end reads. Sequence quality was then assessed using FastQC v0.11.8 and MultiQC v1.6 [2]. Reads were mapped to the EMBL human genome 38, patch release 13, using the splice-aware aligner STAR v2.7.10a [1]. BAM files were sorted and indexed using SAMtools v1.9 [4]. StringTie v2.1.7 was used to generate the gene count matrix [5]. Normalization of fragment counts, PCA, clustering and differential expression analysis were performed in R v4.3.2 with the DESeq2 v1.42.0 package. For visualizing purposes, LFC shrinkage using "`apeglm`" was performed [8]. Unless stated otherwise, an FDR < 0.05 was considered statistically significant in DESeq2 analysis. .

RT-qPCR analysis

We performed transcript level analysis using quantitative real time-PCR. Total mRNA was converted to cDNA using the SuperScript III First-Strand cDNA system (Thermo Fisher, #18091050). PCR analysis of cDNA was

performed using primers specific for human *MCUR1* and the MCU complex subunit genes (see **Supplemental Table S2**). Each reaction was run in triplicate. Amplimer amounts were quantified continuously with the SYBR Green qPCR system (Applied Biosystems, #A46012) using an qTower3 (Analytik Jena). The *GAPDH* signal was used for normalization.

Literature

1. Dobin A, Davis CA, Schlesinger F, Drenkow J, Zaleski C, Jha S, Batut P, Chaisson M, Gingeras TR (2013) STAR: ultrafast universal RNA-seq aligner. Bioinformatics 29:15–21. doi: 10.1093/bioinformatics/bts635

2. Ewels P, Magnusson M, Lundin S, Käller M (2016) MultiQC: summarize analysis results for multiple tools and samples in a single report. Bioinformatics 32:3047–3048. doi: 10.1093/bioinformatics/btw354

3. Ge SX, Jung D, Yao R (2020) ShinyGO: a graphical gene-set enrichment tool for animals and plants. Bioinformatics 36:2628–2629. doi: 10.1093/bioinformatics/btz931

4. Li H, Handsaker B, Wysoker A, Fennell T, Ruan J, Homer N, Marth G, Abecasis G, Durbin R, 1000 Genome Project Data Processing Subgroup (2009) The Sequence Alignment/Map format and SAMtools. Bioinformatics 25:2078–2079. doi: 10.1093/bioinformatics/btp352

5. Pertea M, Pertea GM, Antonescu CM, Chang T-C, Mendell JT, Salzberg SL (2015) StringTie enables improved reconstruction of a transcriptome from RNA-seq reads. Nat Biotechnol 33:290–295. doi: 10.1038/nbt.3122

6. Schwarz JM, Cooper DN, Schuelke M, Seelow D (2014) MutationTaster2: mutation prediction for the deep-sequencing age. Nat Methods 11:361–362. doi: 10.1038/nmeth.2890

7. Seelow D, Schuelke M (2012) HomozygosityMapper2012--bridging the gap between homozygosity mapping and deep sequencing. Nucleic Acids Res 40:W516–W520. doi: 10.1093/nar/gks487

8. Zhu A, Ibrahim JG, Love MI (2019) Heavy-tailed prior distributions for sequence count data: removing the noise and preserving large differences. Bioinformatics 35:2084–2092. doi: 10.1093/bioinformatics/bty895

Supplemental Tables

Supplemental Table S1. Variants found in *MCUR1* and in X-chromosomal genes (*VMA21*, *LAMP2*) in which mutations are known to cause vacuolar myopathies. Segregation of these variants in the family (pedigree see **Supplemental Figure S3A**)

**Gene**	**Position (GRCh37)**	**Location**	**minor allele frequency (gnomad v2.1.1)**	**Index II:1**	**Father I:1**	**Mother I:2**	**Sister II:2**	**Sister II:3**
MCUR1	chr6:13.794.044 T>C	intron	0.30640000	T|T	T|T	T|C	T|T	T|C
	chr6:13.794.110 C>A	intron	0.58400000	A|A	A|A	A|A	A|A	A|A
	chr6:13.799.118 G>A	exon: p(**R268***)	**0.00005351**	**A|A**	**A**|G	**A**|G	**A**|G	**A**|G
	chr6:13.814.338 G>A	no AA change	0.07128000	G|G	G|G	G|A	G|G	G|A
	chr6:13.814.340 T>C	exon: p(S108G)	0.63250000	C|C	C|C	C|C	C|C	C|C
VMA21	chr23:150.565.330 G>C	exon: p(G7R)	0.48330000	C|-	G|-	C|G	C|G	C|C
	chr23:150.565.560 G>T	5'UTR intergenic	0.52400000	T|-	G|-	T|G	T|G	G|G
LAMP2	chr23:119.572.896 C>A	3'UTR	not listed	C|-	C|-	C|A	C|C	C|A
	chr23:119.590.533 T>A	no AA change	0.39130000	A|-	A|-	A|A	A|A	A|A

Supplemental Table S2. Materials used in this study

Antibodies

Antibody directed against	Host	Clonality	Company	Cat#	RRID
Beta Tubulin	Rabbit	Polyclonal	Abcam	ab6046	AB_2210370
EMRE (SMDT1)	Rabbit	Polyclonal	Abcam	ab157387	N/A
GAPDH (6C5)	Mouse	Monoclonal	Invitrogen	AM4300	AB_437392
Goat anti-Rabbit IgG Alexa Fluor Plus 488	Goat	Polyclonal	Invitrogen	A32731	AB_2633280
Goat Anti-Rabbit IgG Peroxidase Conjugate	Goat	Polyclonal	Calbiochem	DC03L	AB_437852
MAP1LC3B	Rabbit	Polyclonal	Sigma-Aldrich	L7543	AB_796155
MCU (D2Z3B)	Rabbit	Monoclonal	Cell signaling	14997	AB_2721812
MCUR1	Rabbit	Monoclonal	Cell signaling	13706	AB_2749813
Phospho-Pyruvate Dehydrogenase α1 (Ser293) (E4V9L)	Rabbit	Monoclonal	Cell signaling	37115	AB_2923272
Pyruvate Dehydrogenase (C54G1)	Rabbit	Monoclonal	Cell signaling	3205	AB_2162926
Vinculin	Mouse	Monoclonal	Sigma-Aldrich	V9131	AB_477629

Recombinant DNA	Use	Source	RRID
pcDNA3.2-V5-mtLuc		This study	Addgene 219666
pCMV CEPIA2mt	green fluorescent organelle-entrapped protein indicator for the mtCa^{2+} measurement	Addgene, deposited by the Iino Lab	Addgene 58218
CMV-R-GECO1	red intensiometric genetically encoded Ca^{2+}-indicator for optical imaging	Addgene, deposited by the Campbell Lab	Addgene 32444

Oligonucleotides	Sequence	Use
MCUR1_Seq-F	5'-GAAAATACAGCTAACACTGATAAACA-3'	Sanger Sequencing
MCUR1_Seq-R	5'-CAGGAGGCTCAGGTCCATTT-3'	Sanger Sequencing
MCUR1_qPCR-F	5'-AAGCTTGTCTGCTGGATCCC-3'	qPCR Analysis
MCUR1_qPCR-R	5'-CATGGTTTTGAGGCCAGCAA-3'	qPCR Analysis
MCU_ qPCR-F	5'-GCTGTCAGTTCACACTCAAG-3'	qPCR Analysis
MCU_qPCR-R	5'-AAGGAGGAGGAGGTCTATTC-3'	qPCR Analysis
EMRE_qPCR-F	5'-CGGGACACTCATTAGCAAG-3'	qPCR Analysis
EMRE_qPCR-R	5'-CTGATAGGGAAGGCAGAGA-3'	qPCR Analysis
GAPDH_qPCR-F	5'-CTGGTAAAGTGGATATTGTTGCCAT-3'	qPCR Analysis
GAPDH_qPCR-R	5'-TGGAATCATATTGGAACATGTAAACC-3'	qPCR Analysis
