# Supplementary material for: Biallelic MCUR1 nonsense mutation associated with vacuolar myopathy and altered mitochondrial calcium signaling
Source: Acta Neuropathol Commun. 2026 May 5;14:107. doi: 10.1186/s40478-026-02313-y (PMC13162457; doi:10.1186/s40478-026-02313-y)
Supplement: Supplementary file 2 — Supplementary Material 2 [file 40478_2026_2313_MOESM2_ESM.pdf]

Recessive nonsense *MCUR1* mutation causes vacuolar myopathy and altered mitochondrial calcium signaling

Anna Maria Haschke, Anja von Renesse, Eugenio Graceffo,
Susanne Morales-Gonzalez, Alessandro Prigione, Christoph Hübner,
Werner Stenzel, and Markus Schuelke

Supplemental Material

Uncropped Western blots

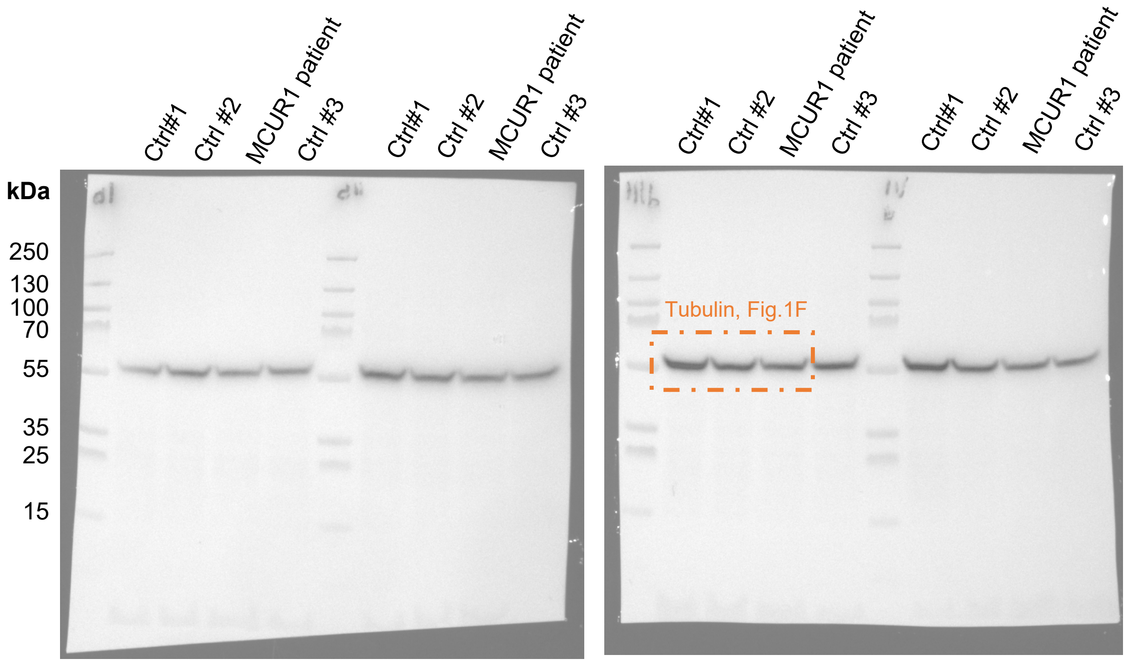

Anti-beta Tubulin antibody - Loading Control (ab6046, AB_2210370)

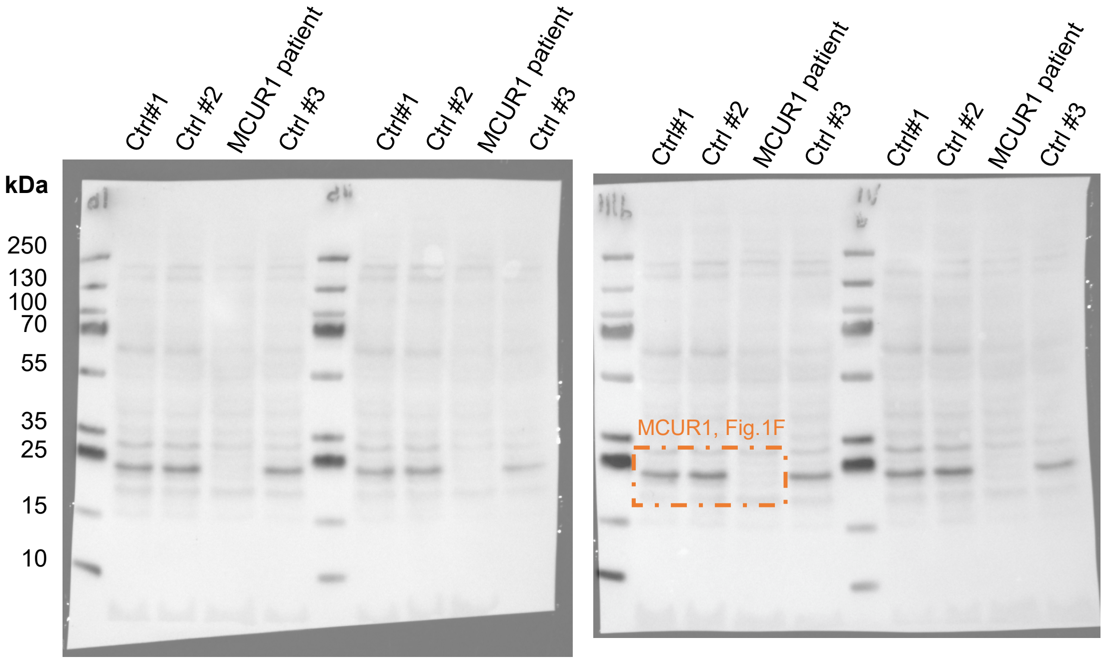

MCUR1 Antibody (#13706, AB_2749813)

Uncropped Western blot images for Figure 1F: 100 µg Protein were loaded on a 4-12% Bis Tris Precast Nupage Precast Gel. PageRuler™ Plus Prestained Protein Ladder (10 to 250 kDa) was used for size reference. Control line #3 had to be excluded from the analysis because the patient was later diagnosed with a mitochondrial disease.

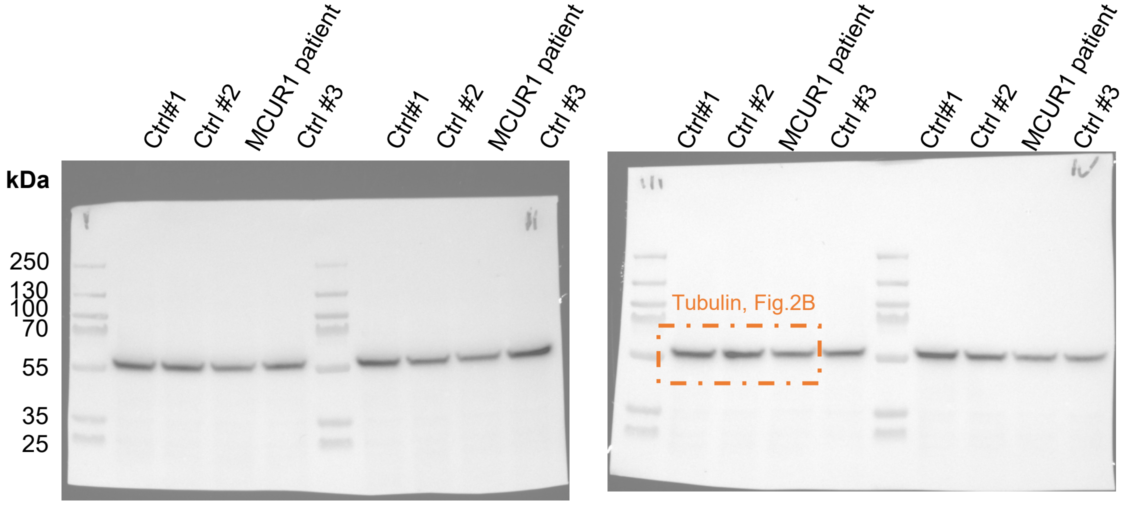

Anti-beta Tubulin antibody - Loading Control (ab6046, AB_2210370)

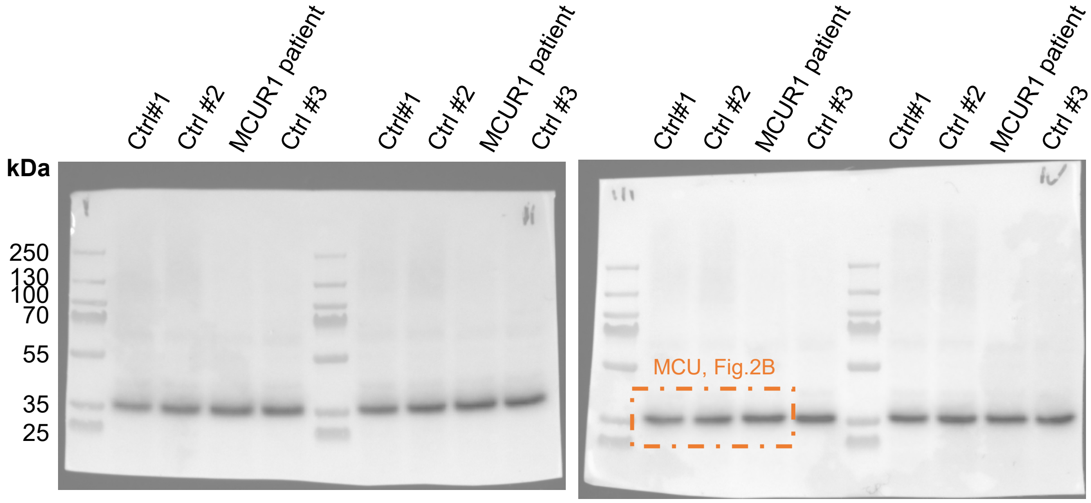

MCU (D2Z3B) Rabbit mAb (#14997, AB_2721812)

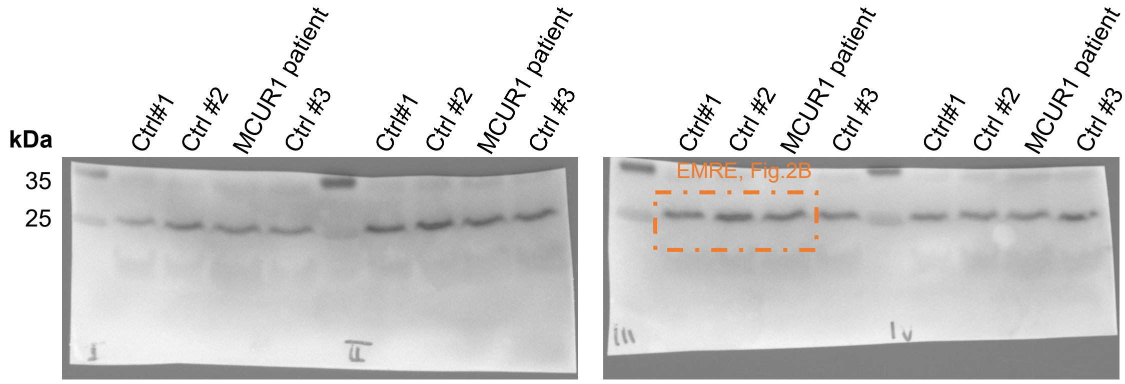

Anti-EMRE antibody (ab157387)

Uncropped Western blot images for Figure 2B: 60 µg Protein were loaded on a 4-12% Bis Tris Precast Nupage Precast Gel. PageRuler™ Plus Prestained Protein Ladder (10 to 250 kDa) was used for size reference. Control line #3 had to be excluded from the analysis because the patient was later diagnosed with a mitochondrial disease.

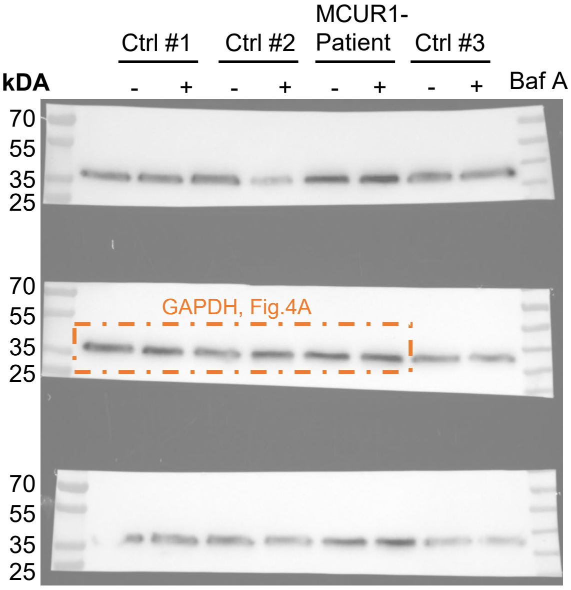

Anti-GAPDH (6C5) antibody (AB_437392)

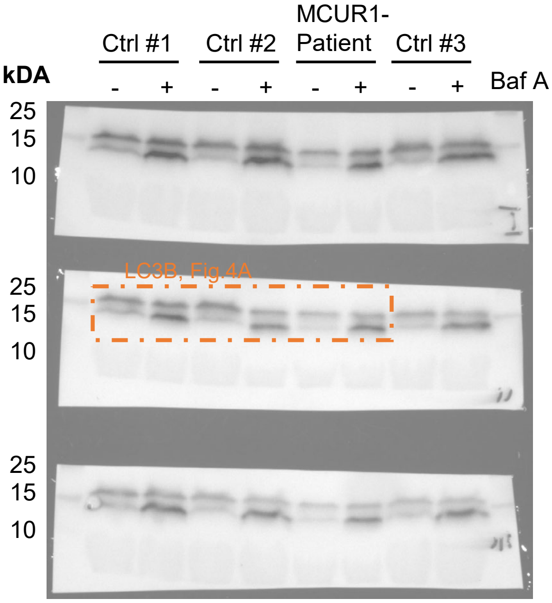

Anti-MAP1LC3B antibody (AB_796155)

Uncopped Western blot images for Figure 4A: 65 µg Protein were loaded on a Biorad Gele 4-20% TBX Gel. PageRuler™ Plus Prestained Protein Ladder (10 to 250 kDa) was used for size reference. Control line #3 had to be excluded from the analysis because the patient was later diagnosed with a mitochondrial disease.
